# Exploring the function and pathogenicity of Goatpox virus N1L gene using recombinant vaccinia virus Tiantan strain

**DOI:** 10.3389/fvets.2025.1622506

**Published:** 2025-07-07

**Authors:** Jialiang Xin, Yaqi Shi, Qian Du, Yanjuan Liao, Zhongyi Zhao, Jingshan Bi, Jiuqing Peng, Sheng Cheng, Chuanhuo Hu, Min Zheng

**Affiliations:** ^1^Key Laboratory of Animal Disease and Human Health of Sichuan Province, College of Veterinary Medicine, Sichuan Agricultural University, Chengdu, China; ^2^Guangxi Center for Animal Disease Control and Prevention, Nanning, China; ^3^Yunnan Animal Health Supervision Institute, Kunming, China; ^4^College of Animal Science and Technology, Guangxi University, Nanning, China; ^5^Guangxi Key Laboratory of Polysaccharide Materials and Modification, School of Marine Sciences and Biotechnology, Guangxi Minzu University, Nanning, China

**Keywords:** Goatpox virus, N1L, vaccinia virus, Tian Tan strain, biological functions

## Abstract

The N1L gene is a well-characterized virulence factor in the poxvirus family; however, its functional role in Goatpox virus (GTPV) remains poorly understood. To elucidate the biological significance of the GTPV N1L gene (gN1L), we constructed three recombinant vaccinia virus Tiantan strain (rVVT) using homologous recombination: rVVT-ΔvN1L (deletion of VVT N1L), rVVT-vN1Lr (insertion of enhanced green fluorescent protein, EGFP), and rVVT-gN1L (substitution with gN1L). The biological properties of these recombinant strains were systematically compared with those of wild-type VVT to evaluate the functional role of gN1L. Bioinformatics analysis revealed that the gN1L-encoded protein shares 26.80% homology and 45.10% similarity with the VVT N1L (vN1L)-encoded protein. Notably, the gN1 protein was predicted to be structurally stable, whereas the vN1 protein was classified as unstable. Growth curve assays demonstrated that gN1L significantly enhances VVT replication in BHK-21, HeLa, and PK-15 cells. RNA-seq analysis further suggested that this enhancement is potentially mediated through the PI3K/AKT signaling pathway. *In vitro* and *in vivo* virulence assays indicated that gN1L increases VVT virulence by up to 133-fold, representing a 7.5-fold greater effect compared to vN1L. Additionally, viral load measurements in host tissues revealed that gN1L facilitates VVT traversal across the blood–brain barrier by enhancing its ability to infect glial and endothelial cells. Collectively, these findings provide novel insights into the functional role of gN1L and offer valuable implications for the development of safer attenuated vaccines against GTPV.

## 1 Introduction

Goatpox virus (GTPV), sheeppox virus (SPPV), and lumpy skin disease virus (LSDV) belong to the *Capripoxvirus* genus within the *Poxviridae* family. These enveloped, brick-shaped viruses measure approximately 294 ± 20 nm in length and 262 ± 22 nm in width, and possess large double-stranded DNA genomes (1–3). GTPV and SPPV cause acute, highly contagious diseases in goats and sheep, characterized by fever, skin nodules, respiratory and gastrointestinal lesions, and lymphadenopathy (4). LSDV primarily infects cattle, causing skin nodules and significant economic losses, including reduced milk production, weight loss, abortion, and infertility (5–7). These viruses are globally distributed and pose substantial economic threats due to the high costs of outbreak control and eradication efforts (8–11). Consequently, they are classified as notifiable diseases by the World Organization for Animal Health (WOAH) (4, 12, 13).

Currently, no effective antiviral drugs are available for infections caused by GTPV, SPPV, or LSDV. Vaccination remains the primary strategy for prevention and control. In China, an attenuated vaccine based on the GTPV-AV41 strain is widely used to protect goats against GTPV and SPPV and has also been employed to prevent LSDV outbreaks due to the 96% genetic similarity between GTPV and LSDV. However, this vaccine has limitations, as some vaccinated animals still develop adverse symptoms, including localized lesions, severe secondary infections, and even death (14). These challenges highlight the urgent need for safer and more effective vaccines. A critical step is to deepen our understanding of GTPV gene functions to inform the rational design of next-generation vaccines. However, progress has been hindered by the virus’s narrow host range and the requirement for Biosafety Level 3 (BSL-3) laboratory conditions. As a result, much of our current knowledge about GTPV gene function is extrapolated from studies on Vaccinia virus (VACV), despite the fact that GTPV and VACV belong to different genera. This reliance on VACV as a model raises questions about the accuracy of our understanding of GTPV-specific gene functions, underscoring the need for direct experimental investigations.

The N1L gene is a well-documented virulence factor within the poxvirus family. Studies have shown that VACV lacking the N1L gene exhibits significantly reduced pathogenicity compared to the wild-type virus when administered via intracranial injection in mice (15). Similarly, the N1L gene in Ectromelia virus (ECTV) plays a critical role in viral pathogenicity, with N1L-deficient ECTV demonstrating a 1,000-fold reduction in virulence compared to the wild-type virus in subcutaneous mice models (16). Despite its established importance in other poxviruses, the N1L gene in GTPV remains poorly characterized. To date, it is only known that the GTPV N1L gene is encoded by the 135 open reading frame and can inhibit the TNF-*α*- or IL-1β-induced NF-κB pathway (17). However, given that virulence proteins often interact with host cells through multiple mechanisms and pathways, further research is needed to fully elucidate the biological functions of the GTPV N1L (gN1L) gene.

The Tiantan strain of vaccinia virus (VVT) was originally isolated in China in 1926 (18, 19). Through successive passages in monkeys, rabbits, and calves, this strain has been extensively attenuated, resulting in a highly attenuated phenotype with significantly reduced virulence compared to the Western Reserve (WR) strain (20–22). In this study, we constructed a recombinant Vaccinia virus Tiantan strain (rVVT) expressing the gN1L gene using homologous recombination. To comprehensively investigate the biological functions of the gN1L gene, we employed a multidisciplinary approach, including bioinformatics analysis, a series of *in vitro* and *in vivo* experiments, and transcriptomic profiling.

## 2 Materials and methods

### 2.1 Cells, viruses, and animals

BHK-21, HeLa, and PK-15 cells were obtained from the China Center for Type Culture Collection. The VVT strain (GenBank: AF095689) was sourced from the Institute of Virology, Changchun Veterinary Research Institute. Five-week-old male BALB/c mice were purchased from Chengdu Dashuo Experimental Animal Co., Ltd.

### 2.2 Stability analysis of GTPV N1 protein

The GTPV N1 protein (AGZ95457.1) and VVT N1 protein (AAF33880.1) sequences were downloaded from the National Center for Biotechnology Information (NCBI) website.^1^ Sequence alignment was performed using VectorBuilder,^2^ Tand protein stability was assessed using ExPASy ProtParam.^3^

### 2.3 Construction of recombinant viruses

Three recombinant VVT viruses (rVVT) were constructed via homologous recombination: rVVT-ΔvN1L (lacking vN1L, containing EGFP), rVVT-vN1Lr (carrying EGFP), and rVVT-gN1L (deleting vN1L, incorporating gN1L and EGFP). Viral purity was confirmed by RT-qPCR. All four strains were propagated in BHK-21 cells, and their copy numbers and TCID_50_ values were determined (23). Primer sequences are listed in Supplementary Table 1. Relative RNA abundance was quantified using the 2^−ΔΔCt^ method.

### 2.4 Growth characteristics and toxicity evaluation of recombinant viruses *in vitro*

The replication capabilities of the rVVTs were analyzed using growth curves. Cells were infected with rVVTs at 4.23 × 10^3^ copies (0.01 MOI) in six-well plates. DNA levels were measured by qPCR at 2, 4, 6, 8, 10, 12, 24, 36, 48, and 72 h post-infection. Cell viability was assessed using the CCK-8 assay at 12, 24, 48, and 72 h post-infection in 96-well plates. Cytotoxicity was further evaluated at 24 h post-infection in 12-well plates at 4.23 × 10^4^ copies, with crystal violet staining.

### 2.5 Evaluation of toxicity of recombinant viruses *in vivo*

**Figure 1:**
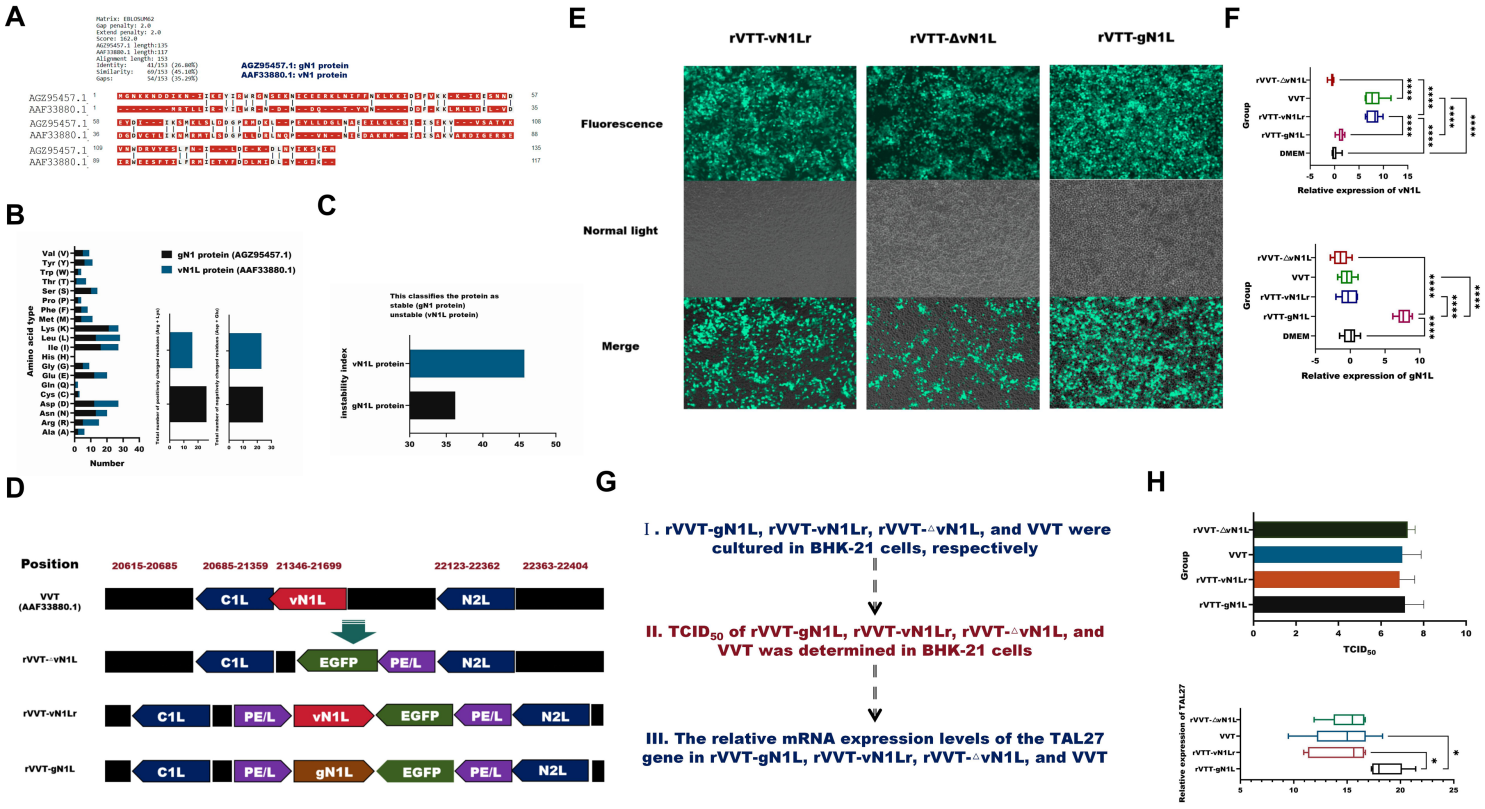
Construction and characterization of recombinant vaccinia virus Tiantan. **(A)** Alignment of amino acid sequences of gN1 and vN1L proteins. **(B)** Amino acid composition analysis of gN1 (AGZ95457.1) and vN1L (AAF33880.1) proteins. **(C)** Comparison of stability indices of gN1 and vN1L proteins. **(D)** Genomic differences between recombinant virus and wild-type VVT. **(E)** Green fluorescent plaques formed by three recombinant viruses in BHK-21 cells under fluorescence microscopy (10 × magnification). **(F)** Purity assessment of viruses by RT-qPCR. **(G)** Expansion culture and quantification of four viruses. **(H)** Determination of TCID_50_ and viral copy number for four viruses. Data are presented as mean ± SD. Statistical significance: ****p < 0.0001, ***p < 0.001, **p < 0.01, *p < 0.05.

BALB/c mice were divided into 25 groups based on intracranial injection doses (Figure 1A). The 50% lethal infectious dose (LD_50_) was determined (23). Pathological analyses were conducted on brain tissues at 10 days post-inoculation (4.23 × 10^4^ to 4.23 × 10^9^ copies), and cytokine levels (IL-6, TNF-*α*, IL-1β) were measured. Viral loads in organs were assessed by RT-qPCR. Primers sequences are shown in Supplementary Table 1. The relative RNA abundance was quantified using the 2−^ΔΔCt^ method.

### 2.6 RNA sequencing

Brain tissue samples were collected from each group of mice, with three pieces of tissue pooled per group to ensure sufficient material for analysis. The pooled brain tissue samples were then sent to Nanning Guotuo Biotechnology Co., Ltd. for total RNA extraction, library construction, and RNA-seq. Differential gene expression DEGs was analyzed from brain samples, with |log2FC| ≥ 1 and a *p*-value < 0.05.

The identified DEGs were analyzed for protein–protein interactions (PPIs) using the STRING database.^4^ To gain insights into the biological functions and pathways associated with the DEGs, Gene Ontology (GO) and Kyoto Encyclopedia of Genes and Genomes (KEGG) pathway enrichment analyses were performed using the DAVID Bioinformatics Resources.^5^ A *p*-value < 0.05 was considered statistically significant for GO functional and KEGG pathway enrichment.

### 2.7 Protein–protein interaction affinity analysis

To verify the accuracy of transcriptome analysis results, we employed RT-qPCR to determine gene expression levels. Primers sequences are shown in Supplementary Table 1. The three-dimensional structures of the gN1 and vN1 proteins were constructed using SwissModel.^6^ Subsequently, the protein structures of Clic4, Gfap, and Himox1 were retrieved from the RCSB PDB database.^7^ ClusPro^8^ was then utilized to analyze the interactions between the two viral proteins and their associated proteins.

### 2.8 Statistical analysis

Data were analyzed using GraphPad Prism 9.5, with significance assessed by unpaired t-tests for two-group comparisons or one-way ANOVA for multiple groups (*p* < 0.05).

## 3 Results

### 3.1 The gN1 protein exhibits greater stability vN1 protein

**Figure 2:**
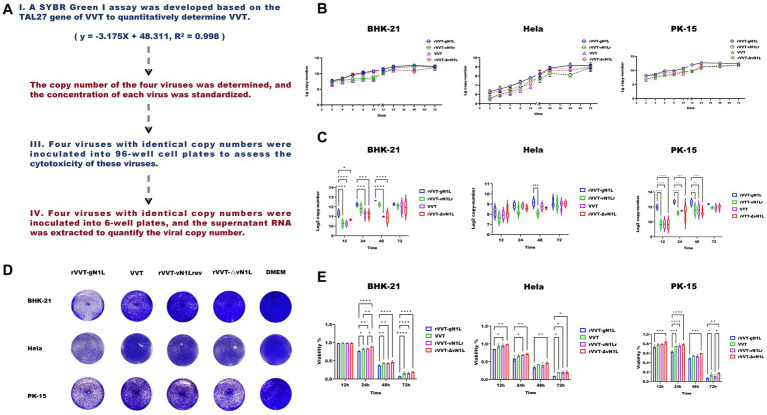
Evaluation of growth ability and virulence of four viruses *in vitro*. **(A)** Schematic of in vitro cell experiment procedures. **(B)** Growth curves of four viruses in BHK-21, HeLa, and PK-15 cells. **(C)** Comparison of viral growth in three cell types at 12 h, 24 h, 48 h, and 72 h. **(D)** Cytotoxicity (%) measured in BHK-21, HeLa, and PK-15 cells at 12 h, 24 h, 48 h, and 72 h. **(E)** Viral plaques stained with crystal violet in three cell types infected with four viruses. Data are presented as mean ± SD. Statistical significance: ****p < 0.0001, ***p < 0.001, **p < 0.01, *p < 0.05.

**Figure 3:**
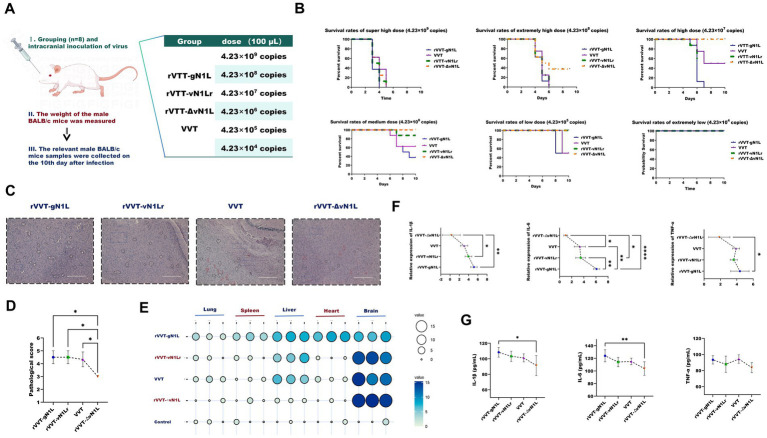
Evaluation of virulence differences among four viruses *in vivo*. **(A)** Experimental procedure and grouping of mice for *in vivo* studies. **(B)** Survival rates of BALB/c mice infected intranasally or intracranially with rVVT-gN1L, rVVT-vN1L, rVVT-ΔvN1L, or VVT at doses of 4.23 × 10^4^ to 4.23 × 10^9^ copies. Survival was monitored for 10 days. **(C)** Histopathological analysis of brain tissues from mice infected with rVVT-gN1L, rVVT-vN1L, VVT, or rVVT-ΔvN1L (4.23 × 10^6^ copies). Red circles: Large triangular neurons; Black circles: Vacuolated cells; Blue squares: Interstitial tissue. **(D)** Pathological scores of brain tissues from mice infected with different viruses. **(E)** Viral load in mouse organs quantified by RT-qPCR. **(F)** Levels of IL-6, TNF-*α*, and IL-1β in brain tissues measured by RT-qPCR. **(G)** Levels of IL-6, TNF-α, and IL-1β in brain tissues measured by ELISA. Data are presented as mean ± SD. Statistical significance: ****p < 0.0001, ***p < 0.001, **p < 0.01, *p < 0.05.

Sequence alignment revealed that gN1 and vN1 proteins shared 26.80% identity and 45.10% similarity (Figure 2A). The amino acid compositions of these proteins were distinct, with gN1 exhibiting greater stability than vN1 (Figures 2B,C). Structural differences between the three rVVT viruses and the wild type are depicted in Figure 2D. Recombinant viruses were purified via successive passages and viral fluorescent plaque selection (Figure 2E). RT-qPCR was used to identify the purified viruses. As shown in Figure 3C, rVVT-gN1L amplified gN1L but not vN1L, whereas rVVT-vN1Lr and rVVT-ΔN1L amplified vN1L but not gN1L. The TCID_50_ and viral copy number determination processes are illustrated in Figure 2F. The TCID_50_ values were similar among all four viruses (Figure 2G), but rVVT-gN1L had a significantly higher viral copy number (Figure 2H).

### 3.2 The gN1L gene enhances VVT replication and cytotoxicity *in vitro*

To assess the impact of gN1L on viral proliferation and cytotoxicity, *in vitro* experiments were performed (Figure 3A). Growth curve analysis showed that all four viruses exhibited similar growth patterns in the three cell types tested (Figure 3B). However, rVVT-gN1L had a significantly higher growth rate compared to the other three viruses, which had comparable growth rates (Figure 3C). Crystal violet staining revealed that plaque formation was essentially identical among the four viruses in different cells (Figure 3D), suggesting that the growth advantage of rVVT-gN1L was not significantly greater than that of the other viruses. Cytotoxicity assays indicated that rVVT-gN1L induced the highest level of cytotoxicity, while rVVT-ΔvN1L exhibited the lowest (Figure 3E). rVVT-vN1Lr and VVT displayed similar cytotoxicity levels (Figure 3E).

### 3.3 The gN1L gene enhances VVT toxicity in BALB/c mice

**Figure 4:**
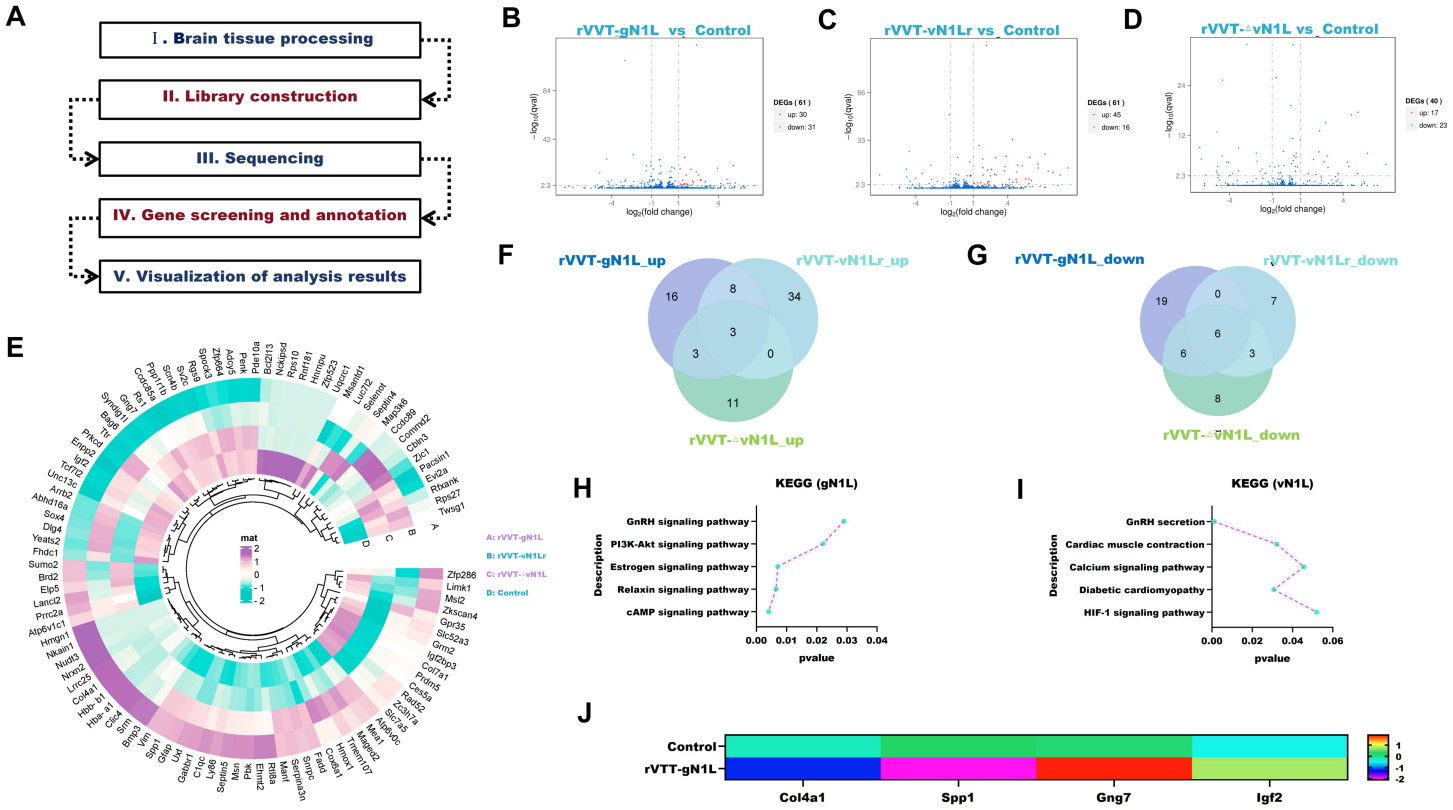
RNA seq analysis to explore the potential pathogenic pathways of gN1L. **(A)** Schematic of the mRNA seq workflow. **(B)** Volcano plot analysis of differentially expressed genes in rVVT-gN1L vs. Control. **(C)** Volcano plot analysis of DEGs in rVVT-vN1Lr vs. Control. **(D)** Volcano plot analysis of DEGs in rVVT-△vN1L vs. Control. **(E)** Heatmap analysis of gene expression profiles. **(F)** Venn diagram analysis of up-regulated genes. **(G)** Venn diagram analysis of down-regulated genes. **(H)** KEGG pathway analysis of gN1L-associated genes. **(I)** KEGG pathway analysis of vN1L-associated genes. **(J)** RT-qPCR validation of gene expression levels in the PI3K/AKT signaling pathway enriched by gN1L. Data are presented as mean ± SD. Statistical significance: ****p < 0.0001, ***p < 0.001, **p < 0.01, *p < 0.05.

To evaluate the pathogenicity of the recombinant viruses, BALB/c mice were intracranially inoculated with different doses of each virus, and their survival rates and pathological indices were monitored (Figure 4A). Except for the DMEM control group, all infected mice exhibited neurological symptoms, including fatigue, convulsions, stiffness, and hair loss, starting on the first day post-inoculation. Mice inoculated with rVVT-gN1L had the earliest mortality and the highest mortality rate (Figure 4B). The median lethal doses (LD_50_) for rVVT-gN1L, rVVT-vN1Lr, VVT, and rVVT-ΔvN1L were calculated as 10^–3.25^/0.1 mL (2.38 × 10^5^ copies), 10^–2.375^/0.1 mL (1.78 × 10^6^ copies), 10^–2.375^/0.1 mL (1.78 × 10^6^ copies), and 10^–1.125^/0.1 mL (3.17 × 10^7^ copies), respectively.

Pathological examination showed that brain tissues from mice inoculated with 4.23 × 10^5^ of rVVT-gN1L exhibited the most severe vacuolar degeneration, while those from rVVT-ΔvN1L showed the least (Figures 4C,D). Viral load analysis revealed that rVVT-gN1L was detectable in all organs, though the brain had the lowest viral load (Figure 4E). In contrast, rVVT-ΔvN1L had the highest concentration in the brain, with only trace amounts in the liver. rVVT-vN1Lr and VVT had comparable viral loads in brain tissue and were also present in the liver and lungs (Figure 4E). RT-qPCR and ELISA analysis showed that the levels of inflammatory factors in the brains of mice infected with rVVT-gN1L were significantly higher than those in other groups (Figure 4F).

### 3.4 The enhanced pathogenicity of gN1L-associated VVT is linked to the PI3K/AKT signaling pathway

The RNA-seq and analysis workflow are shown in Figure 1A. Compared to the control group, the rVVT-gN1L group had 30 upregulated and 31 downregulated genes (Figure 1B). The rVVT-vN1Lr group had 45 upregulated and 16 downregulated genes (Figure 1C), while the rVVT-ΔN1L group had 23 upregulated and 17 downregulated genes (Figure 1D). A heatmap was constructed to highlight 61 differentially expressed genes associated with rVVT-gN1L (Figure 1E). Venn analysis identified target genes associated with gN1L and vN1L. gN1L was responsible for the upregulation of 24 genes and the downregulation of 19 genes (Figures 1F,G), while vN1L led to the upregulation of 42 genes and the downregulation of 7 genes (Figures 1F,G). KEGG pathway enrichment analysis indicated that gN1L-related genes were primarily enriched in the PI3K/AKT signaling pathway, estrogen signaling pathway, and cAMP signaling pathway (Figure 1H). In contrast, vN1L-related genes were associated with GnRH secretion, cardiac muscle contraction, and the HIF-1 signaling pathway (Figure 1I). RT-qPCR validation confirmed the reliability of the transcriptomic data, with expression trends of Col4a1, Spp1, Gng7, and Igf2 consistent with the heatmap (Figure 1J).

### 3.5 gN1L promotes VVT crossing of the host blood–brain barrier

**Figure 5:**
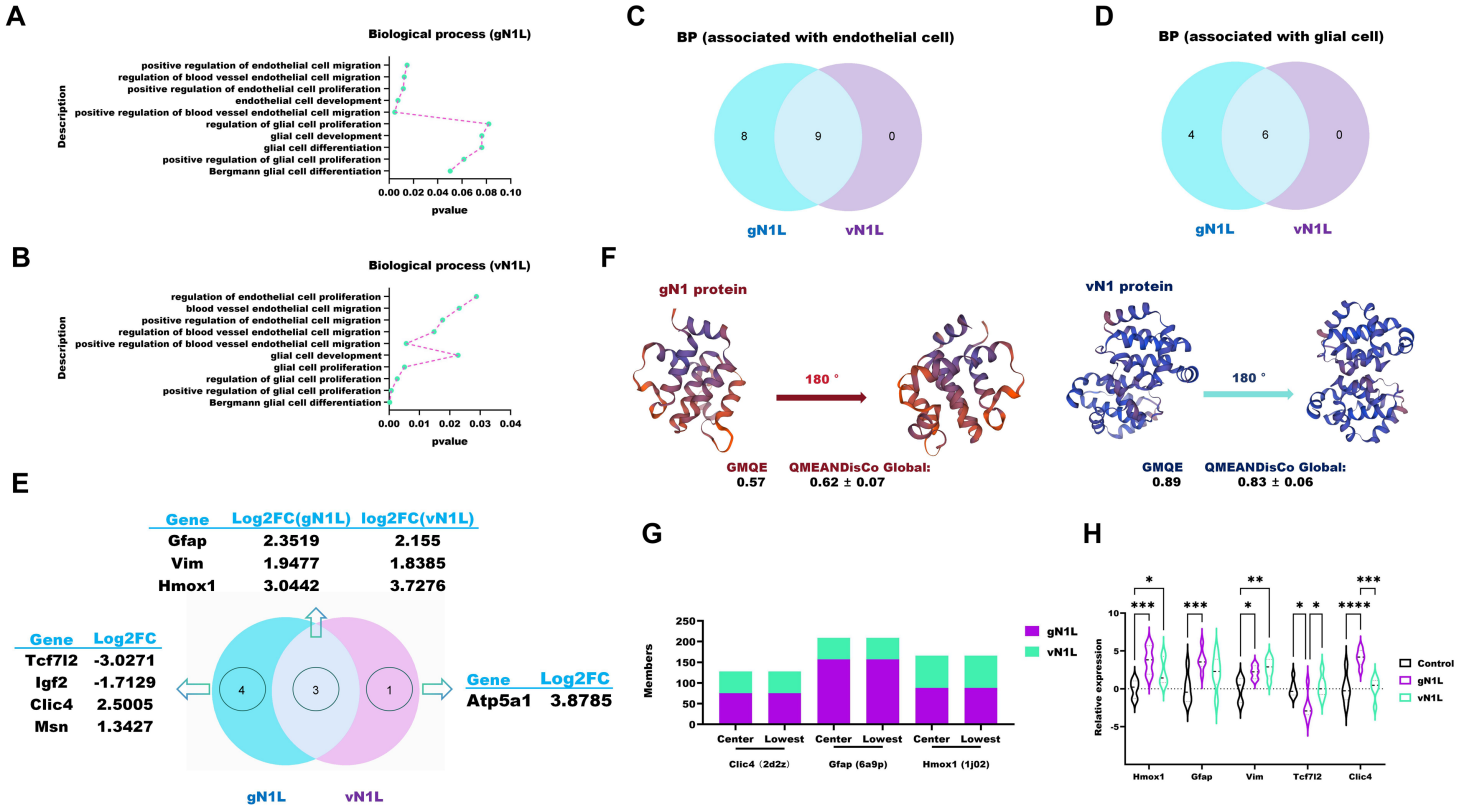
RNA seq-based assessment of the ability of gN1L and vN1L to cross the blood-brain barrier. **(A)** Biological processes related to the blood–brain barrier (BBB) associated with gN1L. **(B)** Biological processes related to the BBB associated with vN1L. **(C)** Venn diagram analysis of endothelial cell-related genes between gN1L and vN1L. **(D)** Venn diagram analysis of glial cell-related genes between gN1L and vN1L. **(E)** Venn diagram analysis of up- and down-regulated genes between gN1L and vN1L. **(F)** 3D structural models of gN1 and vN1 proteins. **(G)** Docking scores of gN1 and vN1 proteins with Clic4, Gfap, and Hmox1. **(H)** RT-qPCR validation of expression levels of related genes. Data are presented as mean ± SD. Statistical significance: ****p < 0.0001, ***p < 0.001, **p < 0.01, *p < 0.05.

To identify genes associated with the blood–brain barrier (BBB), we screened for iterms related to endothelial and glial cells, critical components of the BBB, using Gene Ontology (GO) biological process (BP) annotation (Figures 5A,B). Venn analysis revealed that gN1L-related biological processes included those of vN1L (Figures 5C,D), with significant overlap between the two (Figure 5E).

To assess the structural quality of the encoded proteins, we utilized two evaluation metrics: GMQE (Global Model Quality Estimation) and QMEANDisCo Global. GMQE is an index that evaluates the quality of protein structural models, scored on a scale of 0 to 1, with higher values indicating greater model accuracy (24). QMEANDisCo Global, an extension of QMEAN, evaluates the global quality of protein structure models by calculating the average QMEANDisCo score for each residue (25). Scores range from 0 to 1, with values > 0.6 considered indicative of high-quality models. The three-dimensional structures of the gN1L- and vN1L-encoded proteins were evaluated using GMQE and QMEANDisCo Global metrics (Figure 5F). The GMQE scores for the gN1 and vN1 protein models were 0.57 and 0.89, respectively, while the QMEANDisCo Global scores were 0.62 ± 0.07 and 0.83 ± 0.06, respectively, confirming that the models constructed in this study are of good quality. Compared to the vN1 protein, the gN1 protein formed stronger interactions with the target proteins, as indicated by the higher number of MEMBERS (Figure 5G). MEMBERS refers to the number of similar docking conformations within a cluster, with a larger number indicating greater reliability of the results. RT-qPCR analysis showed that gN1 downregulated the mRNA levels of key genes, including Homx1, Gfap, Vim, Tcf7l2, and Clic4 (Figure 5H).

## 4 Discussion

Attenuated goatpox virus (GTPV) vaccines are crucial for protecting livestock against GTPV, sheeppox virus (SPPV), and lumpy skin disease virus (LSDV) infections (26, 27). However, the severe immune reactions they can induce highlight the urgent need for safer and more effective vaccine alternatives. A deeper understanding of GTPV gene functions and their interactions with host organisms is essential for developing next-generation vaccines with improved safety profiles through rational modification of the viral genome. However, these inferences may not accurately reflect the biological role of gN1L in GTPV. In this study, we used bioinformatics tools to analyze the gN1 and vN1L protein sequences. Our results revealed that the amino acid identity between gN1 and vN1L is only 26.80%, with a similarity of 45.10%. These findings are consistent with a previous study reporting a 20% identity between the N1 protein sequences of VACV strain WR and GTPV AV41 (17), suggesting that the functional roles of these two proteins are not entirely overlapping. Additionally, we observed that the gN1 protein is more stable than the vN1 protein. Given that viral replication and pathogenicity are significantly influenced by the stability and functionality of encoded proteins (28, 29), these findings suggest that gN1 likely plays a more substantial role in enhancing viral pathogenicity within the host.

To investigate the biological function of the gN1 protein, we inserted the gN1L gene into the vaccinia virus Tiantan (VVT) strain. This approach was chosen for two main reasons: first, VVT can be studied under standard laboratory conditions; second, its ability to infect mice significantly reduces experimental costs. Moreover, both VVT and GTPV belong to the poxvirus family, and despite substantial differences in their N1L genes, VVT and GTPV cause very similar symptoms in the host due to many homologous genes between them, such as E4L (30), A4L (31), and A27L (32). These homologous genes result in similar clinical symptoms between the two viruses. By introducing gN1L into VVT, we were able to simulate the gene’s function more accurately by comparing the biological properties of the virus before and after modification.

An intriguing observation emerged during the quantification of viral copy numbers and TCID_50_ values *in vitro*. While the TCID_50_ values for all viruses were similar, the copy number of the virus carrying the gN1L gene was significantly higher than that of the other groups. To further explore the potential role of gN1L in promoting viral replication, we employed an absolute fluorescence quantification method to measure viral copy numbers. After re-inoculating the viruses at a uniform copy number concentration across different cell types, we found that rVVT-gN1L exhibited the highest growth rate, while the growth rates of rVVT-vN1Lr, rVVT-ΔvN1L, and VVT were comparable. This suggests that gN1L enhances VVT proliferation, a finding that contrasts with previous reports indicating that gN1L does not facilitate viral replication (17). This discrepancy likely stems from methodological differences. Previous studies relied on the TCID_50_ method to assess viral growth characteristics (17). While TCID_50_ measures the presence or absence of cytopathic effects (CPE), it does not provide an absolute count of viral particles, as CPE intensity is only loosely correlated with virion quantity (33). In contrast, RT-qPCR offers a more direct and accurate measurement of viral load, enabling precise quantification of viral replication dynamics. In this study, we inoculated three cell types with a uniform copy number of four viruses and measured viral copy numbers in the supernatant at various time points. The results demonstrated that rVVT-gN1L had a higher growth rate in all cell types compared to the other viruses, which exhibited similar growth rates. This confirms that the gN1L gene enhances VVT proliferation, whereas the vN1L gene is non-essential for VVT replication, consistent with prior findings (34). The PI3K/AKT signaling pathway has been implicated in poxvirus replication. When mature VVT particles bind to host cell surface receptors via viral membrane proteins, integrin β1 mediates the activation of PI3K/AKT, facilitating viral endocytosis (35). Furthermore, PI3K/AKT is involved in intracellular viral morphogenesis. For instance, the PI3K inhibitor LY294002 has been shown to stall the immature-virion/immature-virion-with-nucleoid stage in the morphogenic cycles of VACV and CPXV (36). In our transcriptomic analysis, KEGG pathway enrichment revealed that gN1L is associated with the PI3K/AKT signaling pathway, with key enriched genes including Col4a1, Spp1, Gng7, and Igf2. RT-qPCR validation confirmed that the expression trends of these genes aligned with the transcriptomic data. Based on these findings, we hypothesize that gN1L promotes VVT replication by activating the PI3K/AKT signaling pathway.

The gN1 protein not only enhances VVT replication but also significantly amplifies its cytotoxicity. To evaluate the toxicity-promoting effects of gN1L, we conducted both *in vitro* and *in vivo* studies. *In vitro* experiments demonstrated that, at comparable viral copy numbers, rVVT-gN1L exhibited significantly higher cytotoxicity in all three cell types compared to the other viral groups. The cytotoxicity levels of rVVT-vN1Lr and VVT were similar, while rVVT-ΔvN1L showed the lowest toxicity. *In vivo* studies further corroborated these findings, as comparisons of median LD50 values revealed that the presence of gN1L in VVT (with vN1L deleted) increased its toxicity in mice by approximately 133.35-fold, which is about 7.50 times greater than the toxicity observed with vN1L in VVT (with vN1L deleted). These results unequivocally demonstrate that gN1L exerts a significantly stronger promotional effect on VVT cytotoxicity compared to vN1L. Our analysis of viral loads in mice organs revealed a notable pattern. The brain viral load of rVVT-ΔvN1L was significantly higher than that of other groups, while rVVT-gN1L exhibited the lowest brain viral load, despite its robust growth rate in cell cultures. In contrast, rVVT-gN1L was detected in multiple organs, including the heart, lungs, spleen, and liver, whereas rVVT-vN1Lr and VVT were mainly found in the liver and lungs, and rVVT-ΔvN1L was limited to the brain and liver. These findings suggest that the gN1L gene may enhance VVT’s ability to cross the blood–brain barrier (BBB). The BBB, composed of glial cells (astrocytes and microglia), pericytes, and vascular endothelial cells, acts as a critical barrier to prevent pathogens from entering the brain (37, 38). Our transcriptomic analysis showed that gN1L is more involved in biological processes related to glial and vascular endothelial cells than vN1L. Key associated genes included Clic4, Gfap, and Hmox1. Protein–protein docking and RT-qPCR results further validated these observations, confirming their reliability.

## 5 Conclusion

In conclusion, this study demonstrates that the virulence of gN1L against viruses is significantly enhanced. While N1L promotes viral proliferation, this effect does not lead to a qualitative change in viral replication within the host. These findings suggest that N1L may serve as a potential target for the development of attenuated vaccines, offering new insights for optimizing vaccine design strategies.

## Data Availability

The datasets presented in this study can be found in online repositories. The names of the repository/repositories and accession number(s) can be found in the article/Supplementary material.

## Footnotes

^1^ https://www.ncbi.nlm.nih.gov

^2^ https://www.vectorbuilder.cn/tool/sequence-alignment.html

^3^ http://web.expasy.org/protparam/

^4^ https://string-db.org/

^5^ https://david.ncifcrf.gov

^6^ https://swissmodel.expasy.org

^7^ https://www.rcsb.org

^8^ https://cluspro.org

## Supplementary material

SUPPLEMENTARY TABLE 1 The details of primer sequences.
