# Supplementary material for: Exploring the function and pathogenicity of Goatpox virus N1L gene using recombinant vaccinia virus Tiantan strain
Source: Front Vet Sci. 2025 Jul 7;12:1622506. doi: 10.3389/fvets.2025.1622506 (PMC12278286; doi:10.3389/fvets.2025.1622506)
Supplement: SUPPLEMENTARY TABLE 1 — The details of primer sequences. [file Table_1.docx]

| **Primer Name** | **Sequence (5' → 3')** |
| --- | --- |
| IL-6-F | 5’-CTTCTTGGGACTGATGCTGGT-3’ |
| IL-6-R | 5’-GTTGGGAGTGGTATCCTCTGTG-3’ |
| TNF-α-F | 5’-CACGTCGTAGCAAACCACCAA-3’ |
| TNF-α-R | 5’-GTTGGTTGTCTTTGAGATCCAT-3’ |
| IL-1β-F | 5’-AATCTCGCAGCAGCACATCA-3’ |
| IL-1β-R | 5’-GGAAGGTCCACGGGAAAGAC-3’ |
| Gapdh-F | 5′-GCACAGTCAAGGCTGAGAA-3′ |
| Gapdh-R | 5′-GCCAGTAGACTCCACAACATAC-3′ |
| Gng7-F | 5’-TCAGGTACTAACAACGTCGCC-3’ |
| Gng7-R | 5’-CCTTGATGCGTTCGATCCCA-3’ |
| Gfap-F | 5’-CGGAGACGCATCACCTCTG-3’ |
| Gfap-R | 5’-TGGAGGAGTCATTCGAGACAA-3’ |
| Vim-F | 5’-CGTCCACACGCACCTACAG-3’ |
| Vim-R | 5’-GGGGGATGAGGAATAGAGGCT-3’ |
| Hmox1-F | 5’-AGGTACACATCCAAGCCGAGA-3’ |
| Hmox1-R | 5’-CATCACCAGCTTAAAGCCTTCT-3’ |
| Tcf7l2-F | 5’-TCATCACGTACAGCAATGAACA-3’ |
| Tcf7l2-R | 5’-CGACAGCGGGTAATATGGAGAG-3’ |
| Clic4-F | 5’-AAGGCCGGAAGTGATGGTG-3’ |
| Clic4-R | 5’-GGTCAACGGTTGTGACACTGA-3’ |
| Col4a1-F | 5’-ACCATGCCCTTTCTTCTG-3’ |
| Col4a1-R | 5’-GTGCATCACGAAGGAATAGC-3’ |
| Igf2-F | 5’-CGTGGGATGGGTGCTTTCAG-3’ |
| Igf2-R | 5’-AGGGCCACACTAGGCATAGG-3’ |
| Spp1-F | 5’-CATCCCTGTTGCCCAGCTTC-3’ |
| Spp1-R | 5’-TCCGACTGATCGGCACTCTC-3’ |
| vN1L-F | 5’-TTGGTAGATGACGGCGATGT-3’ |
| vN1L-R | 5’-GCGATCATTCGCTTAGCGTC-3’ |
| gN1L-F | 5’-TGGATGATGGTCCAAGGATGG-3’ |
| gN1L-R | 5’-AACGATTCATAAACACGATCCCAA-3’ |
| TAL27-F | 5’-AAACCGTCTTTTCTGGTCTAATA-3’ |
| TAL27-R | 5’-CGTGAAATCGCTCGTAAAAAC-3’ |
